# TRMP, a p53-inducible long noncoding RNA, regulates G1/S cell cycle progression by modulating IRES-dependent p27 translation

**DOI:** 10.1038/s41419-018-0884-3

**Published:** 2018-08-30

**Authors:** Yang Yang, Chenfeng Wang, Kailiang Zhao, Guang Zhang, Decai Wang, Yide Mei

**Affiliations:** Hefei National Laboratory for Physical Sciences at Microscale, The CAS Key Laboratory of Innate Immunity and Chronic Disease, School of Life Sciences, University of Science and Technology of China, Hefei, Anhui China

## Abstract

The tumor suppressor p53 plays a pivotal role in the protection against cancer. Increasing evidence suggests that long noncoding RNA (lncRNA) plays an important role in the regulation of the p53 pathway, however, the detailed mechanisms remain to be further elucidated. In this study, we report a new p53-inducible lncRNA that we termed TRMP (*T*P53-*r*egulated *m*odulator of *p*27). As a direct transcriptional target of p53, TRMP plays an unexpected pro-survival function. Knockdown of TRMP inhibits cell proliferation by inducing a G1 cell cycle arrest. Mechanistically, TRMP suppresses internal ribosomal entry site (IRES)-dependent translation of p27 by competing p27 mRNA for polypyrimidine tract-binding protein 1 (PTBP1) binding. Furthermore, TRMP is able to regulate cell proliferation, G1/S cell cycle progression, and tumor xenograft growth via the inhibition of p27. Taken together, these findings suggest lncRNA as a new layer to fine-tune the p53 response and reveal TRMP as an important downstream effector of p53 activity.

## Introduction

p53 plays a prominent role in the protection against cancer^1–4^. The importance of p53 in tumor suppression is highlighted by the findings that p53 knockout mice are highly prone to spontaneous tumor formation and in more than half of human tumors, p53 is inactivated by its oncogenic mutation^5^. Even in tumors with wild-type p53, the p53 pathway is often disrupted by altered expression of upstream regulators or downstream effectors of p53^6^. Moreover, germline mutations in the p53 gene have been associated with Li–Fraumeni syndrome, a hereditary autosomal dominant cancer disorder^5^. As a tumor suppressor, p53 restrains cell proliferation in response to a variety of cellular stresses including DNA damage and oncogene activation by triggering cell cycle arrest, apoptosis, and senescence. In addition, p53 has been implicated in the regulation of other cellular processes such as cell metabolism, autophagy, and ferroptosis, which may also contribute to its tumor suppressive activity^7–11^.

Due to its strong anti-proliferative effect, p53 is kept at low levels in unstressed cells. The best known negative regulator of p53 is the ubiquitin E3 ligase Mdm2, which targets p53 for rapid proteasome-dependent degradation^12,13^. Upon cellular stresses, such as DNA damage, p53 is stabilized and binds to the consensus response elements in the promoter of p53 target genes, thereby leading to their activation^14^. Among them, p21 is a critical cell cycle inhibitor; PUMA and Noxa are potent apoptosis inducers. However, increasing evidence suggests that these well-known p53 target genes may not be fully responsible for mediating p53 function in tumor suppression^15–17^. Therefore, identification and functional investigation of new p53 target genes are of great importance for the comprehensive understanding how p53 exerts its tumor suppressive function.

Although studies on the p53 transcriptional network have largely focused on protein-coding genes, it has been increasingly recognized that p53 is also able to transcriptionally regulate noncoding RNA (ncRNA) species^18–22^. These include microRNAs (miRNAs), and more recently, a number of p53-regulated long noncoding RNAs (lncRNAs) were also identified. LncRNAs are defined as transcripts longer than 200 nucleotides with no protein-coding potential^23^. The current understanding of lncRNAs is relatively limited compared to protein-coding genes, nevertheless, emerging evidence suggests that lncRNAs may serve as critical regulatory molecules that control gene expression and by doing so, they have been implicated in a variety of human diseases including cancer^24–26^. Various mechanisms have been proposed for lncRNAs-regulated gene expression, and one of the important ones is their abilities to form RNA–protein complexes via interacting with various proteins^23^. For instance, p53-inducible lncRNA-ROR is able to interact with heterogeneous nuclear ribonucleoprotein I/polypyrimidine tract-binding protein 1 (hnRNP I/PBPT1), thus leading to the suppression of p53 expression^27^.

As p53 transcriptional targets, lncRNAs have been shown to be involved in both positive and negative regulation of the tumor suppressive function of p53. For example, p53-upregulated lncRNAs including lincRNA-p21, DINO, LED, TP53TG1, Loc285194, TUG1, and NEAT1 exert tumor suppressive activities by inhibiting cell cycle progression, inducing apoptosis, or suppressing oncogene-induced transformation^28–34^. On the contrary, other p53-inducible lncRNAs, such as linc-Pint, PANDA, PURPL, DDSR1, TRINGS, and PVT1, act as pro-survival cellular factors in the face of stress signals^35–40^. These findings demonstrate lncRNA as a critical layer of the regulation of the p53 pathway, and also indicate the functional diversity and complexity of lncRNA in regulating p53 tumor suppressive function. Therefore, further identification and functional elucidation of additional novel p53-responsive lncRNAs could provide new insights into p53-mediated tumor suppression.

In this study, we report a new p53-inducible lncRNA that we named TRMP (*T*P53-*r*egulated *m*odulator of *p*27). TRMP is a direct transcriptional target of p53. We show that TRMP binds to PTBP1 to disrupt its interaction with p27 5′-UTR, thereby leading to the inhibition of internal ribosomal entry site (IRES)-dependent translation of p27. Functionally, TRMP regulates cell proliferation, G1/S cell cycle progression, and tumor xenograft growth via suppression of p27. Collectively, these findings suggest TRMP as a new player that regulates the tumor suppressive function of p53.

## Results

### TRMP is a novel p53-inducible long noncoding RNA

**Fig. 1:**
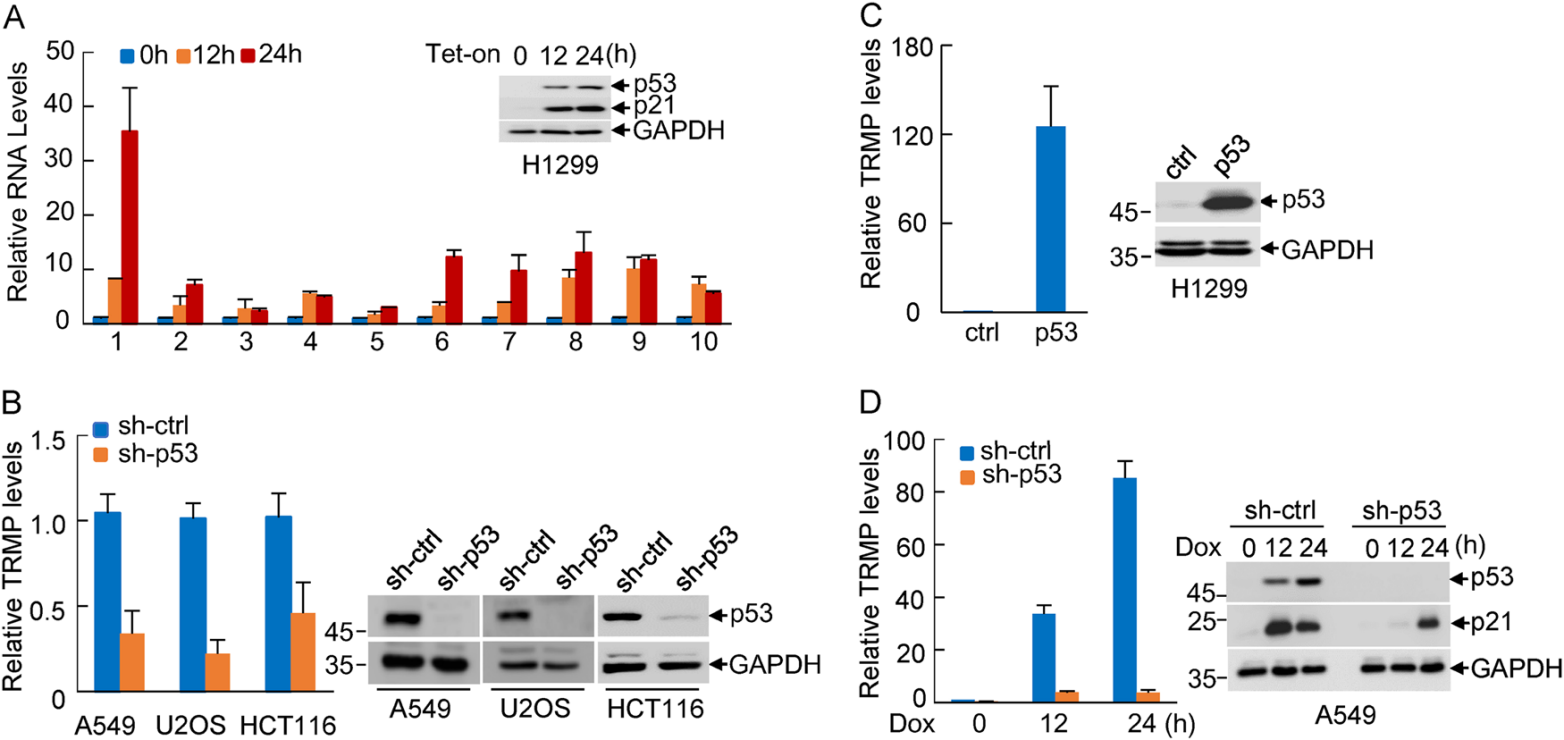
TRMP is up-regulated by p53. **a** H1299 cells with doxycycline-inducible expression of wild-type p53 (H1299-Tet-On-p53) were incubated with doxycycline (1 μg/ml) for the indicated periods of time. Total RNA was then subjected to real-time RT-PCR analysis to examine expression levels of the indicated 10 long non-coding RNAs (lncRNAs). The detailed information of these lncRNAs is shown in Supplementary Table S1. Data shown are mean ± SD of three independent experiments. Cell lysates were also analyzed by western blotting with the indicated antibodies. **b** H1299 cells were infected with lentiviruses expressing wild-type p53. Forty-eight hours later, total RNA was subjected to real-time RT-PCR analysis to examine TRMP levels. Data shown are mean ± SD of three independent experiments. Cell lysates were also analyzed by western blotting. **c** A549, U2OS, and HCT116 cells were infected with lentiviruses expressing either control shRNA or p53 shRNA. Forty-eight hours later, total RNA was subjected to real-time RT-PCR analysis to examine TRMP levels. Data shown are mean ± SD of three independent experiments. Cell lysates were also analyzed by western blotting with the indicated antibodies. **d** A549 cells were infected with lentiviruses expressing either control shRNA or p53 shRNA. Forty-eight hours, cells were treated with doxorubicin (0.5 μg/ml) for the indicated periods of time. Total RNA was then subjected to real-time RT-PCR analysis to examine TRMP levels. Data shown are mean ± SD of three independent experiments. Cell lysates were also analyzed by western blotting

To identify novel lncRNAs that involve in the regulation of p53 function, doxycycline-treated or untreated H1299 cells carrying a p53 tet-on system were used to determine the lncRNA expression profile via microarray analysis. Ten p53-upregulated lncRNAs identified by lncRNA microarray (fold change above 10) were selected for further validation using real-time RT-PCR analysis (Supplementary Table S1). Of these ten lncRNAs, lncRNA-1 (RP11-369C8.1) was found to be most significantly upregulated upon p53 induction (Fig. 1a). Therefore, lncRNA-1 was chosen for further investigation. We should mention that of these ten lncRNAs, lnRNA-9 and lncRNA-10 have been previously reported as p53-inducible lncRNAs^22,41^, indicating the reliability of our lncRNA microarray data. As will be shown in the following sections, lncRNA-1 is able to specifically modulate protein levels of p27, we therefore named it TRMP. TRMP was localized in both the nucleus and cytoplasm (Supplementary Figure S1A). By performing both 5′- and 3′-rapid amplification of cDNA ends (RACE) experiments, we revealed TRMP as a RNA transcript with a molecular size of 834 nt (Supplementary Figure S1B). The bioinformatic analysis revealed that TRMP had no coding potential (Supplementary Figure S1C).

To further verify the inducing effect of p53 on TRMP expression, we knocked down p53 in wild-type p53-containing cell lines such as A549, U2OS, and HCT116. Knockdown of p53 in these cells led to a strong decrease in TRMP expression (Fig. 1b). Conversely, ectopic expression of p53 greatly increased levels of TRMP in H1299 cells (Fig. 1c). Also, treatment with doxorubicin in both A549 and U2OS cells resulted in a time-dependent increase in TRMP levels, which was accompanied by the expected induction of p53 and its target gene p21 (Fig. 1d, Supplementary Figure S1D). However, when p53 was knocked down in these cells, the effect of doxorubicin on TRMP expression was greatly diminished (Fig. 1d, Supplementary Figure S1D). Moreover, TRMP was also upregulated upon p53 induction in primary IMR90 cells (Supplementary Figure S1E). Collectively, these results demonstrate TRMP as a p53-inducible lncRNA.

### TRMP is a direct transcriptional target of p53

**Fig. 2:**
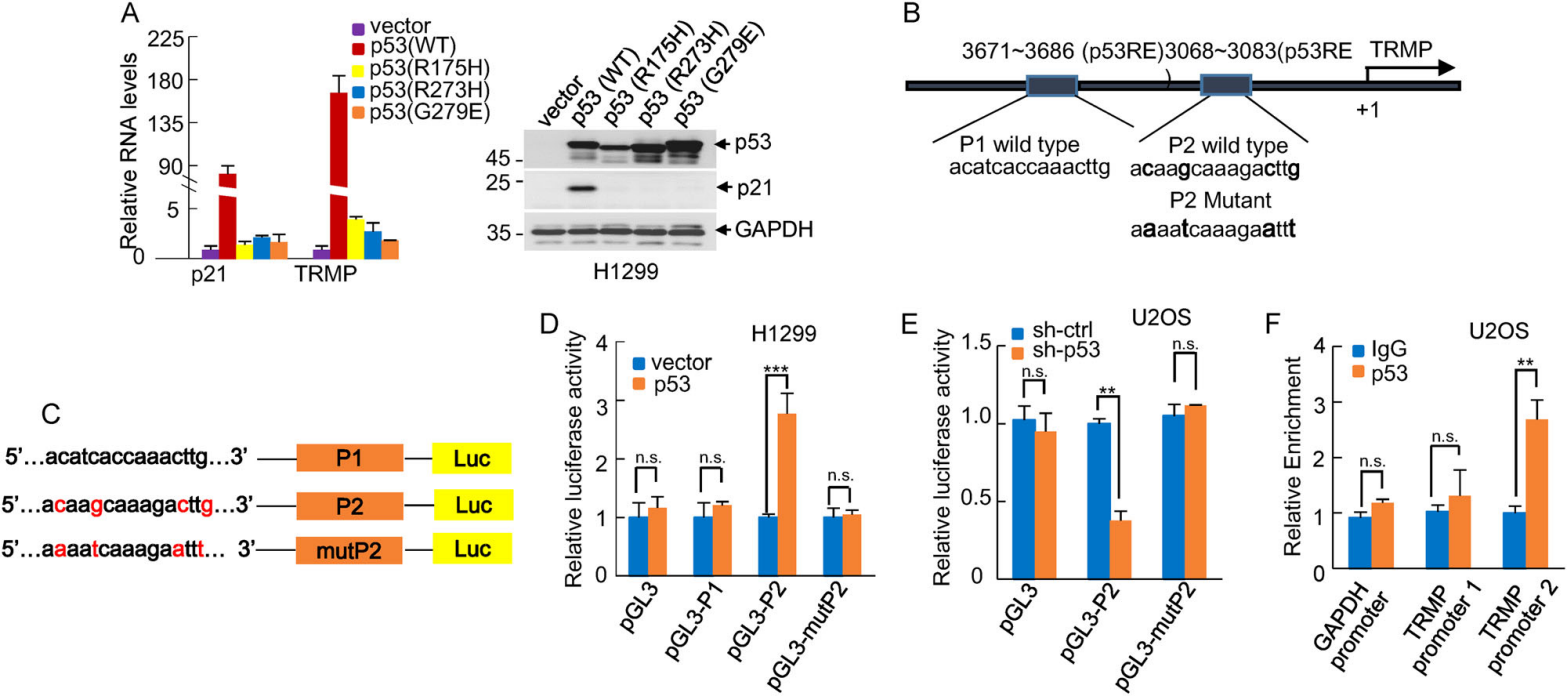
TRMP is a direct transcriptional target of p53. **a** H1299 cells were infected with lentiviruses expressing wild-type p53 or the indicated p53 mutant forms. Forty-eight hours later, total RNA was subjected to real-time RT-PCR analysis to examine TRMP levels. Data shown are mean ± SD of three independent experiments. Cell lysates were also analyzed by western blotting. **b** Schematic illustration of two putative p53 binding sites (P1 and P2) in *TRMP* gene promoter. **c** Shown are the pGL3-based wild-type and mutant reporter constructs used for luciferase assay. **d** H1299 cells were co-transfected with either control vector, Flag-p53, or together with the reporter constructs in the indicated combination. Twenty-four hours after transfection, the reporter activity was measured and plotted after normalizing with respect to Renilla luciferase activity. Data shown are mean ± SD of three independent experiments. *** and n.s. indicate *p* < 0.001 and no significance, respectively. **e** U2OS cells expressing either control shRNA or p53 shRNA were co-transfected with the indicated reporter constructs and Renilla luciferase plasmid. Twenty-four hours after transfection, the reporter activity was measured and plotted after normalizing with respect to Renilla luciferase activity. Data shown are mean ± SD of three independent experiments. ** and n.s. indicate *p* < 0.01 and no significance, respectively. **f** Lysates from U2OS cells were subjected to ChIP assay using anti-p53 antibody or an isotope-matched control IgG. ChIP products were amplified by real-time RT-PCR with the indicated pairs of primers. Data shown are mean ± SD of three independent experiments. ** and n.s. indicate *p* < 0.01 and no significance, respectively

To examine whether p53 transcriptionally upregulates TRMP, we used three tumor-associated p53 mutants (R175H, R273H, and G279E), which have lost the p53 transcriptional activity^5^. Unlike wild-type p53, all the examined three tumor-associated p53 mutants showed minimal effects on TRMP and p21 expression (Fig. 2a), indicating that p53 indeed regulates TRMP at the transcriptional level. We next sought to determine whether TRMP is a direct target gene of p53. We inspected 4000 base pair genomic DNA sequence upstream of transcription start site (TSS) of *TRMP* gene using the JASPAR database. Two putative p53 binding sites (P1 and P2) were identified in this region (Fig. 2b). We therefore evaluated whether these two p53 binding sites confer p53-dependent activity. DNA fragments containing wild-type or mutant binding sites were inserted into the promoter region of a pGL3-based luciferase reporter plasmid (Fig. 2c). Luciferase expression from the reporter containing the P2 site, but not from the reporter containing the P1 site, was induced by ectopic expression of p53 and decreased by knockdown of p53 (Fig. 2d, e). By contrast, the mutant P2 site showed no response to either p53 induction or p53 knockdown (Fig. 2d, e). The subsequent chromatin immunoprecipitation (ChIP) assays also verified the association of p53 and the chromatin fragment containing the P2 site (Fig. 2f). Together, these data suggest that TRMP is a bona fide transcriptional target of p53.

### TRMP regulates cell proliferation and G1/S cell cycle progression

**Fig. 3:**
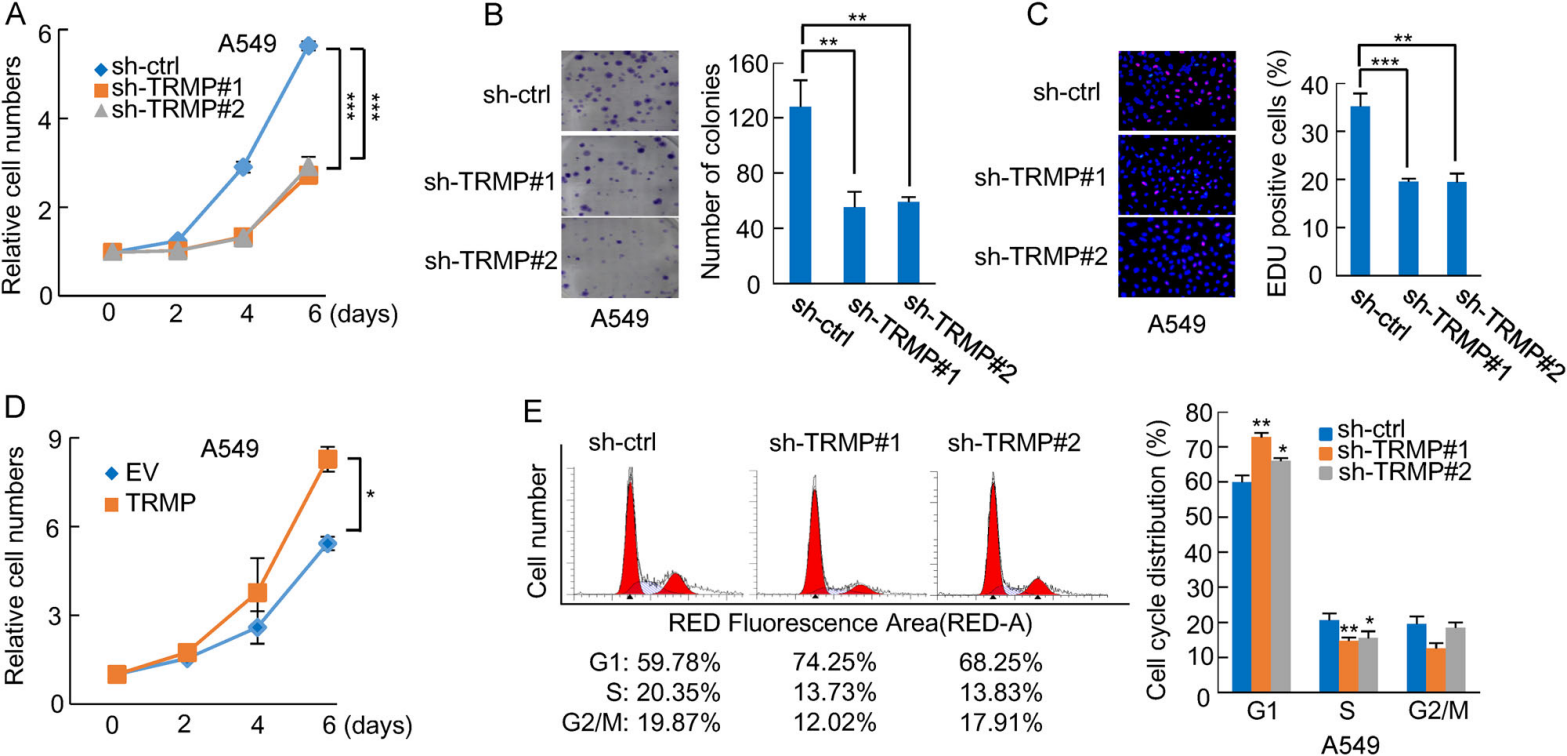
TRMP regulates cell proliferation and G1/S cell cycle progression. **a** A549 cells were infected with lentiviruses expressing control shRNA, TRMP shRNA#1, or TRMP shRNA#2. Forty-eight hours after infection, cells were plated (day 0), and cell numbers were counted at the indicated time points. Data shown are mean ± SD of three independent experiments. *** indicates *p* < 0.001. The knockdown efficiency of TRMP is shown in Supplementary Figure S2A. **b** A549 cells were infected with lentiviruses expressing control shRNA, TRMP shRNA#1, or TRMP shRNA#2. Forty-eight hours after infection, 200 cells were plated and cultured for an additional 10 days. The colonies were then stained with crystal violet. The shown images are representative of three independent experiments. Data shown are mean ± SD (*n* = 3). ** indicates *p* < 0.01. **c** A549 cells were infected with lentiviruses expressing control shRNA, TRMP shRNA#1, or TRMP shRNA#2. Forty-eight hours later, cells were subjected to EdU staining. The nuclei were also visualized by Hoechst 33342 staining. The shown images are representative of three independent experiments. The percentage of proliferating cells was expressed as the ratio of EdU-positive cells to total Hoechst 33342-positive cells. Data shown are mean ± SD (*n* = 3). ** and *** indicate *p* < 0.01 and *p* < 0.001, respectively. **d** A549 cells were infected with lentiviruses expressing either control RNA or TRMP. Forty-eight hours after infection, cells were plated (day 0), and cell numbers were counted at the indicated time points. Data shown are mean ± SD (*n* = 3). * indicates *p* < 0.05. The successful overexpression of TRMP is shown in Supplementary Figure S2F. **e** A549 cells were infected with lentiviruses expressing control shRNA, TRMP shRNA#1, or TRMP shRNA#2. Seventy-two hours after infection, cells were subjected to flow cytometry analysis to evaluate cell cycle distribution. Data shown are mean ± SD of three independent experiments. * and ** indicate *p* < 0.05 and *p* < 0.01, respectively

To investigate the functional role of TRMP, we knocked down TRMP in A549 and U2OS cells. To avoid off-target effect, two different TRMP-targeting shRNAs (sh-TRMP#1 and sh-TRMP#2) were used. Both of these two TRMP shRNAs were able to efficiently knock down TRMP expression (Supplementary Figure S2A). Knockdown of TRMP resulted in a dramatic decrease in the proliferation of both A549 and U2OS cells (Fig. 3a, Supplementary Figures S2A–S2C). In addition, TRMP knockdown strongly reduced the number of colonies from A549 cells (Fig. 3b). The number of EdU-positive cells was also decreased by TRMP knockdown (Fig. 3c, Supplementary Figures S2D and S2E). These data suggest that TRMP promotes cell proliferation. In support of this, ectopic expression of TRMP indeed enhanced the proliferation of both A549 and U2OS cells (Fig. 3d, Supplementary Figures S2F–S2H). We next sought to determine how TRMP regulates cell proliferation. We performed flow cytometry analysis to evaluate the effect of TRMP on cell cycle distribution. Knockdown of TRMP led to an increase in the percentage of cells in the G1 phase and a decrease in the percentage of cells in S phase (Fig. 3e, Supplementary Figure S2I), indicating that TRMP knockdown inhibits G1/S cell cycle progression. Given that TRMP is upregulated by p53, we asked whether TRMP could regulate p53-induced cell cycle arrest. P53-deficient H1299 cells expressing control shRNA or TRMP shRNA were infected with lentiviruses expressing exogenous p53, followed by flow cytometry analysis. The results showed that the inhibitory effect of p53 on G1/S cell cycle progression was greatly enhanced by TRMP knockdown in H1299 cells (Supplementary Figure S2J), indicating that TRMP indeed regulates p53-initiated G1 cell cycle arrest. Taken together, these data suggest that TRMP regulates cell proliferation via modulating G1/S cell cycle progression.

### TRMP specifically regulates protein expression of p27

**Fig. 4:**
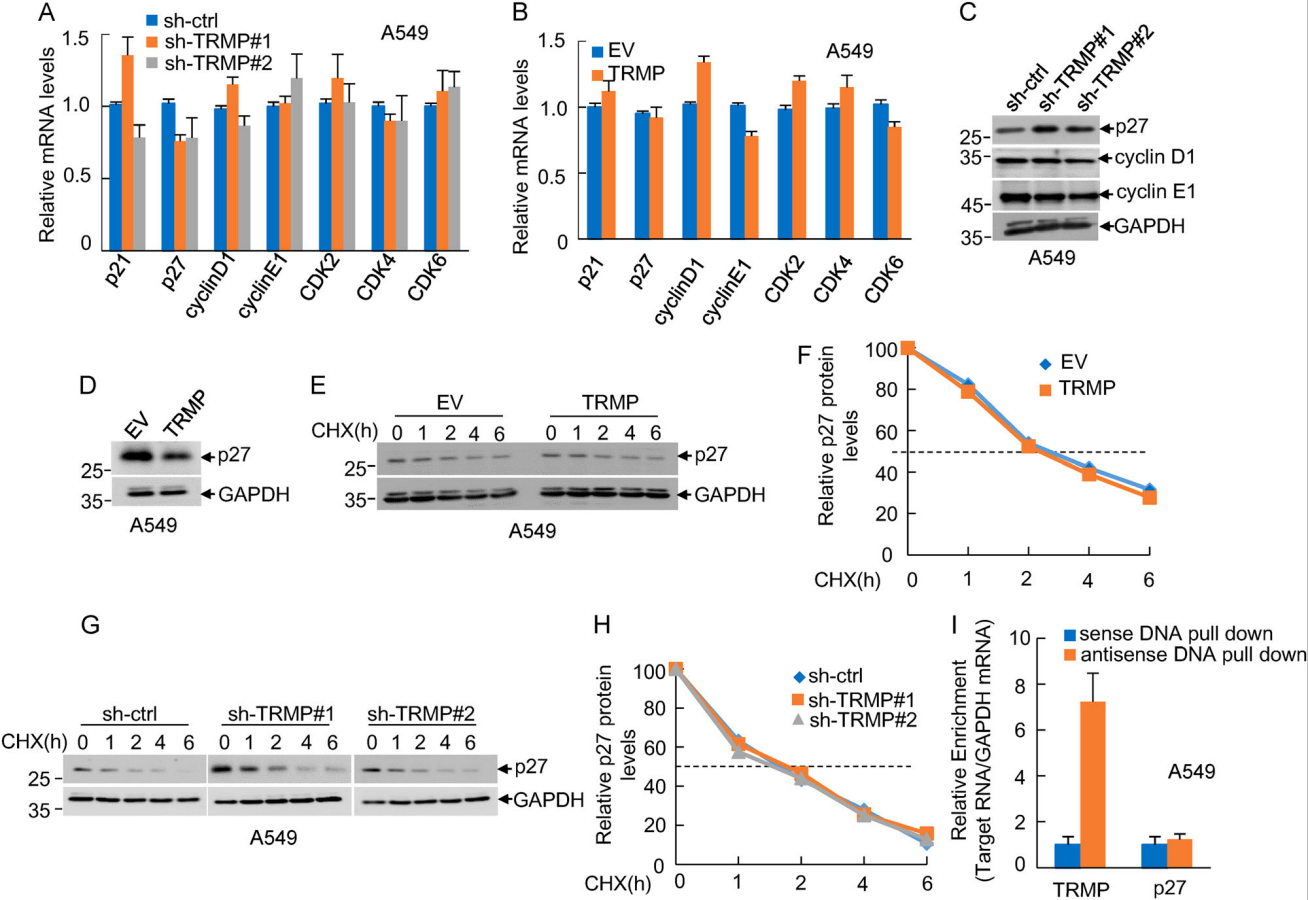
TRMP specifically modulates protein levels of p27. **a** A549 cells were infected with lentiviruses expressing control shRNA, TRMP shRNA#1, or TRMP shRNA#2. Forty-eight hours after infection, total RNA was subjected to real-time RT-PCR analysis to examine mRNA levels of the indicated genes. Data shown are mean ± SD (*n* = 3). **b** A549 cells were infected with lentiviruses expressing either control RNA or TRMP. Forty-eight hours after infection, total RNA was subjected to real-time RT-PCR analysis to examine mRNA levels of the indicated genes. Data shown are mean ± SD (*n* = 3). **c** A549 cells were infected with lentiviruses expressing control shRNA, TRMP shRNA#1, or TRMP shRNA#2. Forty-eight hours after infection, cell lysates were analyzed by western blotting with the indicated antibodies. The band intensities were quantified by using ImageJ software. The ratio of p27 to GAPDH is presented in Supplementary Figure S3C. **d** A549 cells were infected with lentiviruses expressing either control RNA or TRMP. Forty-eight hours after infection, cell lysates were analyzed by western blotting with anti-p27 antibody. The band intensities were quantified by using ImageJ software. The ratio of p27 to GAPDH is presented in Supplementary Figure S3D. **e**, **f** A549 cells were infected with lentiviruses expressing either control RNA or TRMP. **e** Forty-eight hours after infection, cells were treated with cycloheximide (CHX, 20 μg/ml) for the indicated periods of time. Cell lysates were then analyzed by western blotting. **f** The band intensities were quantified by using ImageJ software. The ratio of p27 to GAPDH was also presented. **g**, **h** A549 cells were infected with lentiviruses expressing control shRNA, TRMP shRNA#1, or TRMP shRNA#2. **g** Forty-eight hours after infection, cells were treated with cycloheximide (CHX, 20 μg/ml) for the indicated periods of time. Cell lysates were then analyzed by western blotting to determine p27 protein half-life. **h** The band intensities were quantified by using ImageJ software. The ratio of p27 to GAPDH was also presented. **i** Lysates from A549 cells were incubated with either sense or antisense biotin-labeled DNA oligomers corresponding to TRMP, followed by the pull-down experiments using streptavidin-coated beads. The pull-downed complexes were subjected to real-time RT-PCR analysis to detect p27 mRNA levels

To explore the mechanism whereby TRMP regulates G1/S cell cycle progression, we examined whether TRMP could modulate the mRNA levels of G1-related cyclins and CDKs genes, such as p21, p27, cyclin D1, cyclin E1, CDK2, CDK4, and CDK6. The results showed that the mRNA levels of these G1 cell cycle-related genes were not affected by either knockdown or ectopic expression of TRMP (Fig. 4a, b, Supplementary Figures S3A and S3B). However, intriguingly, knockdown of TRMP increased, whereas ectopic expression of TRMP decreased the protein levels of p27 (Fig. 4c, d, Supplementary Figures S3C–S3H), implying that TRMP regulates p27 protein expression at the post-transcriptional level. We next determined how TRMP regulates p27 protein levels. It has been previously shown that p27 protein undergoes rapid turnover via the ubiquitin–proteasome-dependent degradation pathway^42^. We therefore evaluated whether TRMP could regulate the half-life of p27. Consistent with the previous reports^42^, p27 was revealed as a labile protein with a half-life of approximately 2 h. Nevertheless, the half-life of p27 was not affected by either knockdown or ectopic expression of TRMP in both A549 and U2OS cells (Fig. 4e–h, Supplementary Figures S3I–S3L), indicating that TRMP does not interfere with p27 protein turnover. We went on to test whether TRMP could regulate p27 protein expression via interacting with p27 mRNA. The results from the biotinylated oligonucleotide pull-down experiment showed no interaction between TRMP and p27 mRNA (Fig. 4i).

### TRMP suppresses p27 protein levels by competing p27 mRNA for PTBP1 binding

**Fig. 5:**
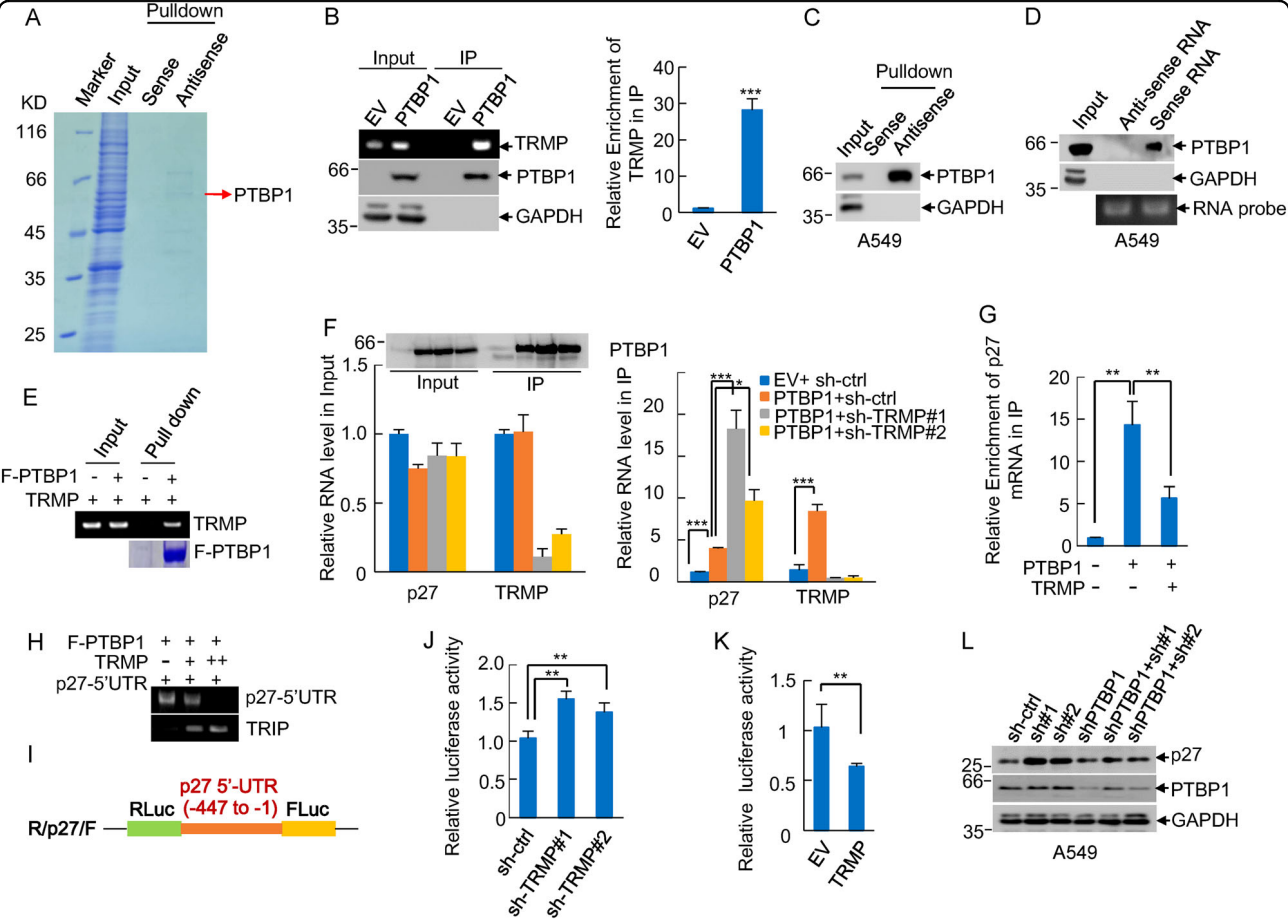
TRMP regulates p27 protein levels by competing p27 mRNA for PTBP1 binding. **a** Lysates from A549 cells were incubated with either sense or antisense biotin-labeled DNA oligomers corresponding to TRMP, followed by the pull-down experiments using streptavidin-coated beads. The pull-downed proteins were separated by SDS-PAGE and visualized by Coomassie brilliant blue staining. The separated proteins were analyzed by mass spectrometry. PTBP1 was identified as a potential TRMP-binding protein. The PTBP1 peptide sequences obtained by MS are shown in Supplementary Figure S4A. **b** Lysates from HEK293T cells expressing Flag or Flag-PTBP1 were immunoprecipitated with anti-Flag antibody. Eluted products from the immunoprecipitates were used for examining TRMP and PTBP1 levels by semi-quantitative RT-PCR and western blot analyses, respectively. The same eluted products were also subjected to real-time RT-PCR analysis to examine TRMP levels. Data shown are mean ± SD (*n* = 3). **c** Lysates from A549 cells were incubated with either sense or antisense biotin-labeled DNA oligomers corresponding to TRMP, followed by the pull-down experiments using streptavidin-coated beads. The pull-downed complexes were analyzed by western blotting with anti-PTBP1 and anti-GAPDH antibodies. The same complexes were also subjected to real-time RT-PCR analysis to confirm the enrichment of TRMP by the antisense DNA oligomer, as shown in Supplementary Figure S4B. **d** Lysates from A549 cells were incubated with in vitro synthesized biotin-labeled TRMP or its antisense RNA, followed by the pull-down experiments using streptavidin-coated beads. The pull-downed complexes were analyzed by western blotting with anti-PTBP1 and anti-GAPDH antibodies. **e** In vitro synthesized TRMP was incubated with purified recombinant Flag-PTBP1 bound with M2 beads. After incubation and extensive washing, the beads-bound RNAs were eluted as templates for RT-PCR analysis. **f** HEK293T cells expressing control shRNA, TRMP shRNA#1, or TRMP shRNA#2 were transfected with or without Flag-PTBP1 as indicated. Twenty-four hours after transfection, cell lysates were immunoprecipitated with anti-Flag antibody. RNAs present in the input and immunoprecipitates were analyzed by real-time RT-PCR. Data shown are mean ± SD (*n* = 3). The input and immunoprecipitates were also analyzed by western blotting with anti-Flag antibody. * and *** indicate *p* < 0.05 and *p* < 0.001, respectively. **g** HEK293T cells were transfected with plasmids expressing Flag-PTBP1 and TRMP RNA in the indicated combination. Twenty-four hours later, cell lysates were immunoprecipitated with anti-Flag antibody, followed by real-time RT-PCR analysis to examine p27 mRNA levels in the immunoprecipitates. Data shown are mean ± SD (*n* = 3). ** indicates *p* < 0.01. **h** Purified Flag-PTBP1 proteins bound with M2 beads were incubated with in vitro synthesized p27 5′UTR (−447 to −1) in the absence or presence of different doses of in vitro transcribed TRMP. After incubation and extensive washing, the beads-bound RNAs were eluted as templates for RT-PCR analysis. **i** Shown is the luciferase reporter plasmid containing the p27 5′-UTR (−447 to −1) used in this study. **j** HEK293T cells expressing control shRNA, TRMP shRNA#1, or TRMP shRNA#2 were transfected with the p27 5′-UTR luciferase reporter plasmid. Twenty-four hours after transfection, the reporter activity was measured and plotted after normalizing with respect to Renilla luciferase activity. Data shown are mean ± SD (*n* = 3). ** indicates *p* < 0.01. **k** HEK293T cells over-expressing either control RNA or TRMP were transfected with the p27 5′-UTR luciferase reporter plasmid. Twenty-four hours after transfection, the reporter activity was measured and plotted after normalizing with respect to Renilla luciferase activity. Data shown are mean ± SD (*n* = 3). ** indicates *p* < 0.01. **l** A549 cells were infected with lentiviruses encoding control shRNA, PTBP1 shRNA, TRMP shRNA#1, and TRMP shRNA#2 in the indicated combinations. Forty-eight hours after infection, cell lysates were subjected to western blot analysis with anti-p27, anti-PTBP1, and anti-GAPDH antibodies. The band intensities were quantified by using ImageJ software. The ratio of p27 to GAPDH is presented in Supplementary Figure S4D

It has been well recognized that lncRNAs may exert their functions through binding to their target proteins^23^. We therefore sought to identify novel TRMP-binding proteins and determine whether TRMP regulates p27 levels via the interacting proteins. Proteins pulled down by sense and antisense DNA oligomers corresponding to TRMP were separated by SDS-PAGE and analyzed by mass spectrometry. A unique protein band with a molecular weight of approximately 60 kDa present in the TRMP antisense DNA oligomer precipitates was identified as PTBP1 (Fig. 5a, Supplementary Figure S4A). This attracted our particular attention because of the following reasons. PTBP1 is a well-known RNA binding protein. Although a major role of PTBP1 is involved in mRNA splicing^43^, PTBP1 can also serve as a regulator of gene expression by binding to 5′-UTR of the target mRNAs and promoting their IRES-dependent translation^44,45^. More importantly, it has been previously shown that PTBP1 enhances p27 translation via binding to the p27 5′-UTR IRES site^45^. We therefore hypothesized that TRMP may regulate p27 translation though competing with p27 mRNA for PTBP1 binding.

To further validate the interaction of PTBP1 with TRMP, an immunoprecipitation-reverse transcription polymerase chain reaction (IP-RT-PCR) assay was performed. TRMP was readily detected in the Flag-PTBP1 immunoprecipitates, but not in the control immunoprecipitates (Fig. 5b). The PTBP1–TRMP interaction was also verified by affinity pull-down of endogenous TRMP using a biotin-labeled antisense DNA oligomer against TRMP (Fig. 5c, Supplementary Figure S4B). In addition, the in vitro biotin-labeled TRMP, but not its antisense RNA, was able to pull down endogenous PTBP1 protein (Fig. 5d, Supplementary Figure S4C). Furthermore, an in vitro binding assay with purified PTBP1 and in vitro transcribed TRMP showed that PTBP1 is directly associated with TRMP (Fig. 5e). Together, these data indicate PTBP1 as an interacting partner for TRMP.

We next examined whether TRMP regulates the binding of PTBP1 to p27 mRNA. The results showed that knockdown of TRMP increased, whereas ectopic expression of TRMP decreased the binding of PTBP1 to p27 mRNA (Fig. 5f, g). More importantly, an in vitro binding assay showed that TRMP was indeed capable of inhibiting the interaction between PTBP1 and p27 5′-UTR in a dose-dependent manner (Fig. 5h), suggesting TRMP competes with p27 mRNA for PTBP1 binding. To evaluate the effect of TRMP on p27 5′-UTR IRES activity, the 5′-UTR (−447 to −1) of p27 was cloned into a dual luciferase reporter plasmid (Fig. 5i). We were able to show that TRMP indeed inhibited the p27 5′-UTR-driven firefly luciferase activity. For example, TRMP knockdown caused an approximately 50% increase of the luciferase activity, while TRMP ectopic expression decreased the activity by over 40% (Fig. 5j, k). To further determine the role of PTBP1 in TRMP-regulated p27 protein expression, TRMP and PTBP1 were knocked down individually or combined in A549 and U2OS cells. The results consistently showed that knockdown of TRMP resulted in increased protein expression of p27, however, which was greatly reversed by the simultaneous knockdown of PTBP1 (Fig. 5l, Supplementary Figures S4D–S4F), indicating that TRMP regulates p27 protein expression via PTBP1. Taken together, these data suggest that TRMP regulates p27 protein levels by competing p27 mRNA for PTBP1 binding.

### TRMP regulates cell proliferation, G1/S cell cycle progression, and tumor xenograft growth via p27

**Fig. 6:**
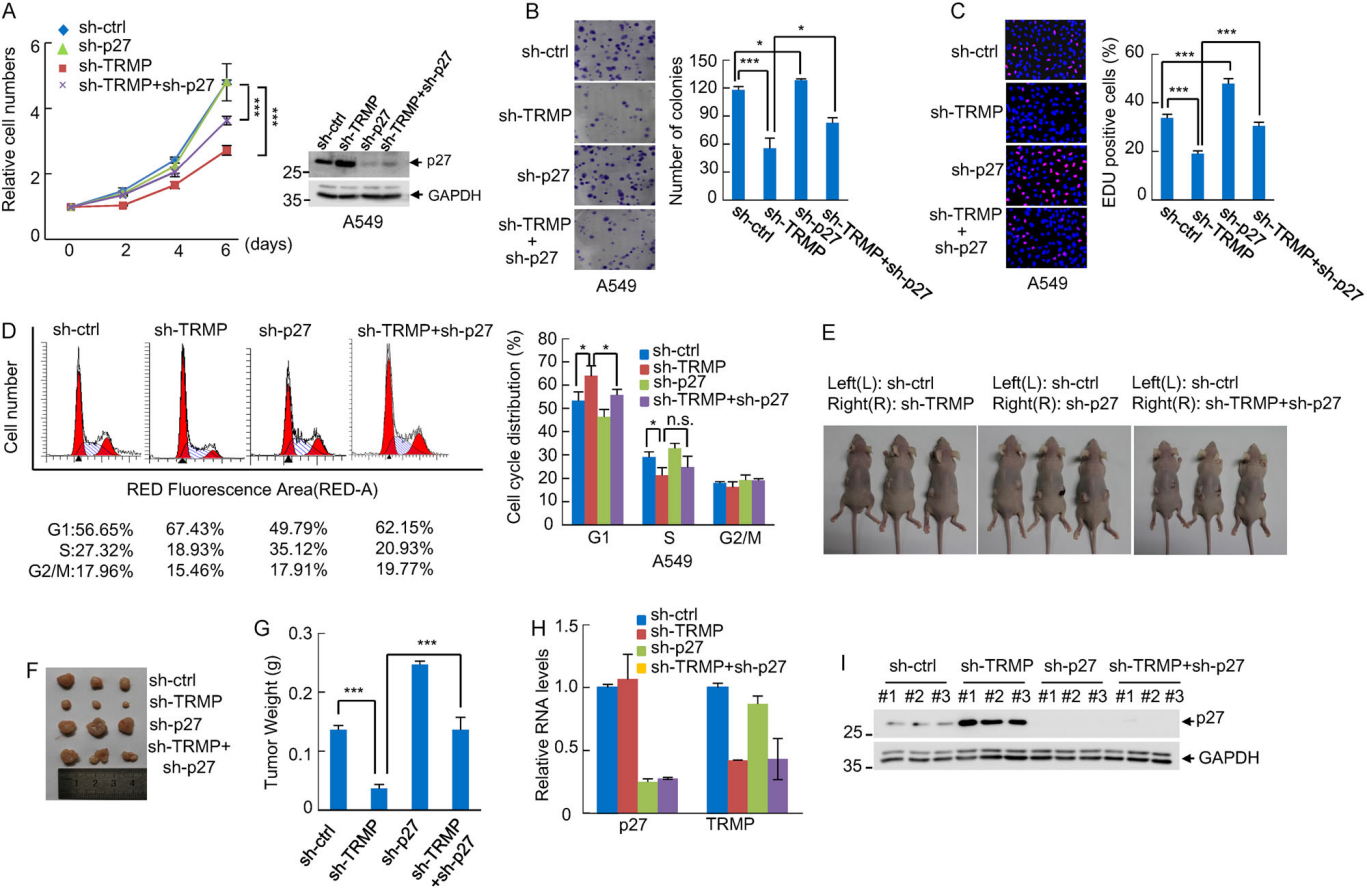
TRMP regulates cell proliferation, G1/S cell cycle progression, and tumor xenograft growth via p27. **a** A549 cells were infected with lentiviruses expressing control shRNA, TRMP shRNA, p27 shRNA, or both TRMP shRNA and p27 shRNA. Forty-eight hours after infection, cells were plated (day 0), and cell numbers were counted at the indicated time points. Data shown are mean ± SD (*n* = 3). Cell lysates were also analyzed by western blotting. *** indicates *p* < 0.001. The knockdown efficiency of TRMP and p27 is shown in Supplementary Figure S5A. **b** A549 cells were infected with lentiviruses expressing control shRNA, TRMP shRNA, p27 shRNA, or both TRMP shRNA and p27 shRNA. Forty-eight hours after infection, 200 cells were plated and cultured for an additional 10 days. The colonies were then stained with crystal violet. The shown images are representative of three independent experiments. Data shown are mean ± SD (*n* = 3). * and *** indicate *p* < 0.05 and *p* < 0.001, respectively. **c** A549 cells were infected with lentiviruses expressing control shRNA, TRMP shRNA, p27 shRNA, or both TRMP shRNA and p27 shRNA. Forty-eight hours later, cells were subjected to EdU staining. The nuclei were also visualized by Hoechst 33342 staining. The shown images are representative of three independent experiments. The percentage of proliferating cells was expressed as the ratio of EdU-positive cells to total Hoechst 33342-positive cells. Data shown are mean ± SD (*n* = 3). *** indicates *p* < 0.001. **d** A549 cells were infected with lentiviruses expressing control shRNA, TRMP shRNA, p27 shRNA, or both TRMP shRNA and p27 shRNA. Seventy-two hours after infection, cells were subjected to flow cytometry analysis to evaluate cell cycle distribution. Data shown are mean ± SD of three independent experiments. * and n.s. indicate *p* < 0.05 and no significance, respectively. **e**–**i** 2 × 10^6^ A549 cells expressing control shRNA, TRMP shRNA, p27 shRNA, or both TRMP shRNA and p27 shRNA were individually injected to the left flank (L) and right flank (R) of the indicated mice. Three weeks after injection, the mice were sacrificed and photographed (**e**). The excised xenografts in each group were compared (**f**). Tumor weights were also shown. *** indicates *p* < 0.001 (**g**). The excised xenografts were homogenized for both RNA and protein extraction. RNA extracts were subjected to real-time RT-PCR analysis. Data shown are mean ± SD of three independent experiments (**h**). Protein extracts were also analyzed by western blotting with anti-p27 and anti-GAPDH antibodies (**i**)

Given the inhibitory effect of TRMP on p27 protein expression as we mentioned above, we next evaluated whether TRMP regulates cell proliferation and G1/S cell cycle progression via p27. TRMP and p27 were knocked down individually or combined in A549 cells. Knockdown of TRMP effectively inhibited the proliferation of A549 cells, which could be partially restored by the simultaneous knockdown of p27 (Fig. 6a, Supplementary Figure S5A). Similar results were also obtained in U2OS cells (Supplementary Figures S5B and S5C). TRMP knockdown consistently reduced the numbers of colonies and EdU-positive cells. However, these effects were significantly reduced by the simultaneous p27 knockdown (Fig. 6b, c, Supplementary Figures S5D and S5E). Moreover, p27 knockdown was also shown to markedly reverse the effect of TRMP on G1/S cell cycle progression (Fig. 6d, Supplementary Figure S5F). Collectively, these data suggest that the effect of TRMP on cell proliferation and G1/S cell cycle progression is mediated, at least partially, by p27.

To investigate whether the TRMP–p27 axis could regulate tumor growth in vivo, we used a xenograft mouse model. A549 cells stably expressing either control shRNA, TRMP shRNA, p27 shRNA, or both TRMP shRNA and p27 shRNA were injected subcutaneously into the dorsal flanks of the nude mice. Three weeks after injection, mice were sacrificed and tumors were excised. The excised xenografts were also homogenized for examining expression levels of TRMP and p27. The results showed that TRMP knockdown A549 cells gave rise to tumors with smaller sizes and lower weights compared to control cells (Fig. 6e–g). Accompanied with the decreased tumor growth by TRMP knockdown, p27 levels were dramatically elevated in xenografts from TRMP knockdown A549 cells (Fig. 6h, i), indicating that TMRP may regulate tumor growth via p27. In support of this, the tumor growth inhibition by TRMP knockdown was substantially reversed by the simultaneous knockdown of p27 (Fig. 6e–i). Taken together, these results indicate an important role of the TRMP–p27 axis in the regulation of tumor xenograft growth.

## Discussion

It has been increasingly recognized that lncRNA plays an important role in the regulation of the p53 response^18,19^. Therefore, identification and functional analysis of new p53-regulated lncRNA is of great importance to the understanding of p53 biology. The current work reveals TRMP as a new p53-inducible lncRNA. TRMP suppresses p27 protein levels by competing with p27 mRNA for PTBP1 binding, and regulates cell proliferation, G1/S cell cycle progression, and tumor xenograft growth by the inhibition of p27.

As a potent tumor suppressor, p53 expression levels are strictly restrained in unstressed cells, which is mainly achieved by the principle E3 ubiquitin ligase Mdm2. Intriguingly, Mdm2 itself is a direct p53 transcriptional target. This establishes an important negative feedback loop to avoid an uncontrolled p53 response^2,12,13^. In addition to the protein-coding gene Mdm2, several p53-inducible lncRNAs are also involved in the negative regulation of the p53 response. For instance, a recently identified p53-upregulated lncRNA PURPL is able to suppress p53 basal levels through interacting with MYBBP1A protein^37^. In addition, PANDA, another p53-regulated lncRNA, interacts with the transcription factor NF-YA to negatively regulate the expression of pro-apoptotic genes during DNA damage^36^. In this study, we show that as a novel p53-inducible lncRNA, TRMP plays a pro-survival function via the inhibition of p27. Collectively, these findings demonstrate lncRNA as an another important layer to fine-tune the p53 response, and also indicate the complexity of the p53 signaling.

p27 belongs to the Cip/Kip family of cyclin-dependent kinase (CDK) inhibitors, and is a negative regulator of cell cycle progression^46^. Due to the potent ability to elicit cell cycle arrest, p27 is considered as a tumor suppressor. In support of this, although mutations in p27 gene are rare, downregulation of p27 protein levels, in particular nuclear-expressed p27, has been associated with a variety of human cancers. In addition, in most tumor types, reduced p27 expression indicates a poorer patient prognosis, making p27 a powerful predictive marker for prognosis in various kinds of cancers including lung, breast, and colon^42,47^. It has been shown that increased expression of p27 typically causes a cell cycle arrest at G1^48^. In consistent with this, we show that accompanied with the enhanced levels of p27, knockdown of TRMP increases the percentage of cells in the G1 phase. In addition, the inhibitory effect of p53 on G1/S cell cycle progression is greatly enhanced by TRMP knockdown. Therefore, TRMP may represent an interesting and new bridge that links p53 to the p27-mediated cell cycle regulation.

It has been shown that regulation of p27 expression occurs at multiple levels. At the transcriptional level, p27 expression is positively regulated by the transcription factors FOXO and MENIN, and inhibited by the oncogenic transcription factor MYC^47,49,50^. At the post-transcriptional level, p27 is subjected to miRNA-mediated regulation or rapid proteasome-dependent degradation initiated by the E3 ubiquitin ligases SCF^SKP2^ and KPC (Kip1 ubiquitination-promoting complex)^51–53^. At the translational level, several RNA binding proteins are able to regulate p27 translation through interacting with the IRES located in its 5′-UTR^45,54^. As an example, via the interaction with p27 5′-UTR, PTBP1 promotes the IRES-dependent translation of p27. In this study, we show that TRMP does not affect the half-life of p27 protein. Intriguingly, TRMP regulates p27 protein expression through modulating PTBP1-mediated p27 translation, as demonstrated by luciferase reporter assays. Mechanistically, TRMP is able to compete with p27 mRNA for PTBP1 binding, as shown by our findings that ectopic expression of TRMP decreased, whereas knockdown of TRMP increased the interaction of PTBP1 with p27 mRNA. This inhibitory effect of TRMP on PTBP1-p27 mRNA binding appears to be direct, as evidenced by an in vitro binding assay, although the detailed mechanisms await for further investigation. It would be interesting to determine whether TRMP and p27 mRNA could interact with PTBP1 at the same binding site. Alternatively, the binding of TRMP to TPBP1 could allosterically inhibit the subsequent p27 mRNA binding. Together with the recent finding of lncRNA UCA1 as a p27-regulatory molecule^55^, our data suggest that lncRNAs may be important, previously unappreciated players in the regulation of the p27 tumor suppressor network. We should also mention that several other lncRNAs have been recently shown to function through interacting with PTBP1. For example, lncRNA Pnky is able to interact with PTBP1 and cooperatively regulate target gene expression, thereby modulating neuronal differentiation of embryonic and postnatal neural stem cells^56^. In addition, lncRNA MEG3 has been reported to induce cholestatic liver injury by interaction with PTBP1 to facilitate Shp mRNA decay^57^. These findings indicate PTBP1 as an important player in the regulation of lncRNA’s cellular function. Given the inhibitory effect of TRMP on p27 expression, it is not surprising that knockdown of TRMP results in decreased cell proliferation in vitro and reduced tumor xenograft growth in vivo. Taken together, our findings suggest that TRMP may exert an oncogenic function to promote tumor growth and implicate TRMP as a new downstream effector of p53 activity.

## Materials and methods

### Reagents and antibodies

The following reagents used in this study were purchased from the indicated sources: cycloheximide (Sigma, 20 μg/ml), doxorubicin (Sigma, 0.5 μg/ml), doxycycline (Sigma, 1 μg/ml), Hoechst 33342 (Sigma, 1 μg/ml), lipofectamine 2000 (Invitrogen), streptavidin-coated agarose beads (Thermo Fisher Scientific), complete EDTA-free protease inhibitor cocktail (Roche Applied Science), antibodies against GAPDH (Santa Cruz, sc-166545, 1:5000), p53 (Santa Cruz, sc-126, 1:1000), p21 (Sigma, #P1484, 1:2000), Flag (Sigma, #F3165, 1:4000), p27 (Abcam, Y236, 1:1000), cyclin D1 (Santa Cruz, sc-753, 1:1000), cyclin E1 (Santa Cruz, sc-481, 1:1000), PTBP1 (Proteintech, 12582-1-AP, 1:1000), HRP-conjugated secondary antibodies against mouse (115-035-062), and rabbit (111-035-144) (Jackson ImmunoResearch, 1:10000).

### Cell culture

A549 and H1299 cell lines were cultured in Roswell Park Memorial Institute (RPMI) 1640 medium (Sigma) supplemented with 10% FBS and antibiotics (GIBCO). HCT116, U2OS, and HEK293T cell lines were maintained in Dulbecco’s modified Eagle’s medium (GIBCO) supplemented with 10% FBS and antibiotics (GIBCO). All cell lines were routinely tested for mycoplasma contamination before they were used for experiments.

### RNA interference

RNA interference was performed as we previously described^58^. To generate lentiviruses expressing the indicated shRNAs, HEK293T cells grown on a 6-cm dish were transfected with 2 μg of shRNA (cloned in PLKO.1) or control vector, 2 μg of pREV, 2 μg of pGag/Pol/PRE and 1 μg of pVSVG. 12 h after transfection, cells were cultured with DMEM medium containing 20% FBS for an additional 24 h. The culture medium containing lentivirus particles was filtered through a 0.45 μm PVDF filter (Millipore) and incubated with the indicated cells supplemented with 8 μg/ml polybrene (Sigma) for 24 h, followed by selection with 2 μg/ml puromycin for another 24 h. The knockdown efficiency was verified by western blot or real-time RT-PCR analysis in each experiment. The shRNA target sequences used in this study are as follows: sh-control, CCTAAGGTTAAGTCGCCCTCG; sh-PTBP1 (human), CCAGCCCATCTACATCCAGTT; sh-p27, GAGCAATGCGCAGGAATAAGG; sh-TRMP#1, GCTGAATACGAAGACAGAACA; sh-TRMP#2, GGGACTATGGAATTTGCAATT.

### Real-time RT-PCR

Total RNA was isolated using Trizol (Invitrogen). 1 μg of RNA was used to synthesize cDNA using PrimeScriptTM RT reagent kit (Takara, DRR037A) according to the manufacturer’s instruction. Real-time PCR was performed using SYBR premix EX Taq (TaKaRa) and analyzed with the StepOnePlus real-time PCR system (Thermo Fisher Scientific). The PCR results, recorded as threshold cycle numbers (Ct), were normalized against an internal control (GAPDH). The expression data were analyzed using the 2^−ΔΔCT^ method described by Livak and Schmittgen^59^. Real-time primer sequences are shown in Supplementary Table S2.

### IP-RT-PCR

IP-RT-PCR was performed as we previously described^60^. Briefly, HEK293T cells were transfected with either control vector or Flag-PTBP1. Twenty-four hours after transfection, cells were lysed in IP lysis buffer (50 mM Tris–HCl, pH 7.4, 150 mM NaCl, 1.5 mM MgCl_2_, 1 mM EDTA, 0.5% NP-40) supplemented with 1× protease inhibitor cocktail, RNase A inhibitor, and DNAse I before centrifugation. Cell lysates were pre-cleared with protein A/G beads (Pierce) before they were incubated with protein A/G beads coated with the indicated antibodies at 4 °C for 8 h. After extensive washing, the beads-bound immunocomplexes were eluted using elution buffer (50 mM Tris–HCl, pH 8.0, 1% SDS, and 10 mM EDTA) at 65 °C for 10 min. To isolate protein-associated RNAs from the eluted immunocomplexes, samples were treated with proteinase K, and RNAs were extracted by phenol/chloroform. Purified RNAs were then subjected to RT-PCR analysis.

### In vitro transcription of biotinylated TRMP and its antisense RNA

To synthesize biotinylated TRMP and its antisense RNA, the DNA template used in the transcription system was generated by RT-PCR using forward and reverse primers containing the T7 RNA polymerase promoter sequence, respectively. PCR products were purified using DNA Gel Extraction kit (AxyPrep). In vitro transcription was then performed using MaxiScript T7 kit (Ambion) in the presence of biotin RNA labeling mix (Roche) according to the instructions provided by the manufacturer.

### Biotin pull-down assay

All the processes were performed in the RNase-free condition. For antisense oligomer affinity pull-down assay, sense or antisense biotin-labeled DNA oligomers corresponding to TRMP (1 μg) were incubated with lysates from 1 × 10^7^ A549 or U2OS cells. One hour after incubation, streptavidin-coated beads (Thermo Fisher Scientific) was added to isolate the RNA–protein complex. For in vitro RNA pull-down assay, 3 μg in vitro synthesized biotin-labeled TRMP or its antisense RNA was incubated with A549 or U2OS cell lysates for 3 h. Streptavidin-coated beads were then used to isolate the RNA–protein complex.

### ChIP assay

The ChIP assay was performed as we previously described^61^. Briefly, U2OS cells were cross-linked with 1% formaldehyde for 10 min. The ChIP assay was performed by using anti-p53 and the ChIP assay kit (Beyotime Biotechnology, Shanghai, China) according to the manufacturer’s instructions. Normal mouse immunoglobulin G (IgG) was also used as a negative control. The bound DNA fragments were subjected to real-time PCR analysis using the specific primers (Supplementary Table S2).

### Luciferase reporter assay

To investigate whether TRMP is transcriptionally regulated by p53, H1299 cells were co-transfected with control vector, Flag-p53, or together with the pGL3-based construct containing wild-type or mutant TRMP promoter plus Renilla luciferase plasmid in the indicated combination. Twenty-four hours later, the reporter activity was measured by using a luciferase assay kit (Promega) and plotted after normalizing with respect to Renilla luciferase activity (mean ± SD).

To determine the effect of TRMP on the IRES activity of p27 5′-UTR, HEK293T cells expressing control shRNA or TRMP shRNA were transfected with the luciferase reporter construct containing p27 5′-UTR (−447 to −1). Twenty-four hours later, the reporter activity was measured by using a luciferase assay kit (Promega) and plotted after normalizing with respect to Renilla luciferase activity. The data are shown as mean ± SD of three independent experiments.

### EdU incorporation assay

The EdU incorporation assay was performed with an EdU Assay Kit (Guangzhou RIBOBIO, Guangzhou, China) according to the manufacturer’s instructions. Briefly, cells were incubated with DMEM medium containing 50 μM EdU for 2 h. The nuclei were also stained with Hoechst 33342 (Sigma, St Louis, MO, USA), and the images were acquired with an Olympus DP73 microscope (Olympus, Tokyo, Japan).

### Cell cycle analysis

A549 and U2OS cells expressing control shRNA, TRMP shRNA#1, TRMP shRNA#2, p27 shRNA, or both TRMP shRNA and p27 shRNA as indicated. Seventy-two hours after infection, cells were harvested and fixed in 70% ethanol at −20 °C overnight, followed by the staining with propidium iodide. Cells were then analyzed for cell cycle distribution by flow cytometry.

### Xenograft mouse model

2 × 10^6^ A549 cells expressing control shRNA, TRMP shRNA, p27 shRNA, or both TRMP shRNA and p27 shRNA were individually injected into the dorsal flanks of 4-week-old male athymic nude mice (Shanghai SLAC Laboratory Animal Co. Ltd.). Mice were used in the experiment at random. Three weeks after injection, the mice were sacrificed and tumors were excised and weighed. During testing the tumors’ weight, the experimentalists were blinded to the information of tumor tissues. The excised tumors were homogenized for RNA and protein extraction. The extracted RNAs and proteins were subjected to real-time RT-PCR and western blot analyses, respectively. Studies on animals were conducted with approval from the Animal Research Ethics Committee of the University of Science and Technology of China.

### Reproducibility

All the data were repeated at least three times. The western blot images were representative of three independent experiments.

### Statistical analysis

Statistical analysis was carried out using Microsoft Excel software and GraphPad Prism to assess differences between experimental groups. Statistical significance was analyzed by Student’s *t*-test and expressed as a *p* value. *p* Values lower than 0.05 were considered to be statistically significant. One asterisk, two asterisks, and three asterisks indicate *p* < 0.05, *p* < 0.01, and *p* < 0.001, respectively. n.s. indicates no significance.

## Electronic supplementary material

Supplementary Information
