# Supplementary material for: TRMP, a p53-inducible long noncoding RNA, regulates G1/S cell cycle progression by modulating IRES-dependent p27 translation
Source: Cell Death Dis. 2018 Aug 30;9(9):886. doi: 10.1038/s41419-018-0884-3 (PMC6117267; doi:10.1038/s41419-018-0884-3)
Supplement: Supplementary file 1 — Supplementary Information [file 41419_2018_884_MOESM1_ESM.pdf]

Supplementary Figure legends

Figure S1 (Related to Figure 1).

(A) Both cytoplasmic and nuclear fractions from A549 cells were analyzed by real-time RT-PCR to determine TRMP localization.

(B) The coding probability of TRMP was analyzed by the Coding Potential Assessment Tool (CPAT).

(C) Shown are the genomic localization and the sequence of TRMP obtained from 5'-RACE (rapid amplification of cDNA ends) and 3'-RACE experiments.

(D) U2OS cells were infected with lentiviruses expressing either control shRNA or p53 shRNA. Forty-eight hours later, cells were treated with doxorubicin (0.5 μg/ml) for the indicated periods of time. Total RNA was then subjected to real-time RT-PCR analysis to examine TRMP levels. Data shown are mean ± SD of three independent experiments. Cell lysates were also analyzed by western blotting.

(E) IMR90 cells were infected with lentiviruses expressing either control or p53 proteins. Forty-eight hours later, total RNA was subjected to real-time RT-PCR analysis to examine TRMP levels. Data shown are mean ± SD of three independent experiments. *** indicates $p<0.001$. Cell lysates were also analyzed by western blotting with the indicated antibodies.

Figure S2 (Related to Figure 3).

(A) The knockdown efficiency of TRMP was verified by real-time RT-PCR analysis for Figure 3A.

(B and C) U2OS cells were infected with lentiviruses expressing control shRNA, TRMP shRNA#1, or TRMP shRNA#2. (B) Forty-eight hours after infection, cells were plated (day 0), and cell numbers were counted at the indicated time points. Data shown are mean ± SD of three independent experiments. *** indicates $p<0.001$. (C) The knockdown efficiency of TRMP was also shown.

(D and E) U2OS cells were infected with lentiviruses expressing control shRNA, TRMP shRNA#1, or TRMP shRNA#2. (D) Forty-eight hours later, cells were subjected to EdU staining. The nuclei were also visualized by Hoechst 33342 staining.

The shown images are representative of three independent experiments. The percentage of proliferating cells was expressed as the ratio of EdU-positive cells to total Hoechst 33342-positive cells. Data shown are mean ± SD (n=3). ** indicates $p<0.01$. (E) The knockdown efficiency of TRMP was also shown.

(F) The successful overexpression of TRMP was determined by real-time RT-PCR analysis for Figure 3D.

(G and H) U2OS cells were infected with lentiviruses expressing either control RNA or TRMP. (G) Forty-eight hours after infection, cells were plated (day 0), and cell numbers were counted at the indicated time points. Data shown are mean ± SD (n=3). * indicates $p<0.05$. (H) The successful overexpression of TRMP was also shown.

(I) U2OS cells were infected with lentiviruses expressing control shRNA, TRMP shRNA#1, or TRMP shRNA#2. Seventy-two hours after infection, cells were subjected to flow cytometry analysis to evaluate cell cycle distribution. Data shown are mean ± SD of three independent experiments. *, ** and *** indicate $p<0.05$, $p<0.01$ and $p<0.001$, respectively.

(J) H1299 cells were infected with lentiviruses expressing control shRNA, p53 proteins, or both TRMP shRNA and p53 proteins as indicated. Forty-eight hours after infection, cells were subjected to flow cytometry analysis to evaluate cell cycle distribution. Data shown are mean ± SD of three independent experiments. ** and *** indicate $p<0.01$ and $p<0.001$, respectively.

Figure S3 (Related to Figure 4).

(A) U2OS cells were infected with lentiviruses expressing control shRNA, TRMP shRNA#1, or TRMP shRNA#2. Forty-eight hours after infection, total RNA was subjected to real-time RT-PCR analysis to examine mRNA levels of the indicated genes. Data shown are mean ± SD (n=3).

(B) U2OS cells were infected with lentiviruses expressing either control RNA or TRMP. Forty-eight hours after infection, total RNA was subjected to real-time RT-PCR analysis to examine mRNA levels of the indicated genes. Data shown are mean ± SD (n=3).

(C) The intensities of bands in Figure 4C were quantified by using ImageJ software. The ratio of p27 to GAPDH was presented.

(D) The intensities of bands in Figure 4D were quantified by using ImageJ software. The ratio of p27 to GAPDH was presented.

(E and F) U2OS cells were infected with lentiviruses expressing control shRNA, TRMP shRNA#1, or TRMP shRNA#2. (E) Forty-eight hours after infection, cell lysates were analyzed by western blotting with the indicated antibodies. (F) The band intensities were quantified by using ImageJ software. The ratio of p27 to GAPDH was also shown.

(G and H) U2OS cells were infected with lentiviruses expressing either control RNA or TRMP. (G) Forty-eight hours after infection, cell lysates were analyzed by western blotting with anti-p27 antibody. (H) The band intensities were quantified by using ImageJ software. The ratio of p27 to GAPDH was also shown.

(I and J) U2OS cells were infected with lentiviruses expressing either control RNA or TRMP. (I) Forty-eight hours after infection, cells were treated with cycloheximide (CHX, 20 μg/ml) for the indicated periods of time. Cell lysates were then analyzed by western blotting. (J) The band intensities were quantified by using ImageJ software. The ratio of p27 to GAPDH was also presented.

(K and L) U2OS cells were infected with lentiviruses expressing control shRNA, TRMP shRNA#1, or TRMP shRNA#2. (K) Forty-eight hours after infection, cells were treated with cycloheximide (CHX, 20 μg/ml) for the indicated periods of time. Cell lysates were then analyzed by western blotting to determine p27 protein half-life. (L) The band intensities were quantified by using ImageJ software. The ratio of p27 to GAPDH was also presented.

Figure S4 (Related to Figure 5).

(A) Shown are the PTBP1 peptide sequences identified by mass spectrometry for Figure 5A.

(B) The enrichment of TRMP by the antisense DNA oligomer against TRMP was verified by real-time RT-PCR analysis for Figure 5C.

(C) Lysates from U2OS cells were incubated with *in vitro* synthesized biotin-labeled TRMP or its antisense RNA, followed by the pull-down experiments using streptavidin-coated beads. The pull-downed complexes were analyzed by western blotting with anti-PTBP1 and anti-GAPDH antibodies.

(D) The intensities of bands in Figure 5L were quantified by using ImageJ software. The ratio of p27 to GAPDH was presented.

(E and F) U2OS cells were infected with lentiviruses encoding control shRNA, PTBP1 shRNA, TRMP shRNA#1, and TRMP shRNA#2 in the indicated combinations. (E) Forty-eight hours after infection, cell lysates were subjected to western blot analysis with anti-p27, anti-PTBP1 and anti-GAPDH antibodies. (F) The band intensities were quantified by using ImageJ software. The ratio of p27 to GAPDH was also shown.

Figure S5 (Related to Figure 6).

(A) The knockdown efficiency of TRMP and p27 was verified by real-time RT-PCR analysis for Figure 6A.

(B and C) U2OS cells were infected with lentiviruses expressing control shRNA, TRMP shRNA, p27 shRNA, or both TRMP shRNA and p27 shRNA. (B) Forty-eight hours after infection, cells were plated (day 0), and cell numbers were counted at the indicated time points. Data shown are mean ± SD (n=3). Cell lysates were also analyzed by western blotting. *** indicates $p<0.001$. (C) The knockdown efficiency of TRMP and p27 was also shown.

(D and E) U2OS cells were infected with lentiviruses expressing control shRNA, TRMP shRNA, p27 shRNA, or both TRMP shRNA and p27 shRNA. (D) Forty-eight hours later, cells were subjected to EdU staining. The nuclei were also visualized by Hoechst 33342 staining. The shown images are representative of three independent experiments. The percentage of proliferating cells was expressed as the ratio of EdU-positive cells to total Hoechst 33342-positive cells. Data shown are mean ± SD (n=3). *** indicates $p<0.001$. (E) The knockdown efficiency of TRMP and p27 was also shown.

(F) U2OS cells were infected with lentiviruses expressing control shRNA, TRMP shRNA, p27 shRNA, or both TRMP shRNA and p27 shRNA. Seventy-two hours after infection, cells were subjected to flow cytometry analysis to evaluate cell cycle distribution. Data shown are mean ± SD of three independent experiments. * indicates $p<0.05$.

A

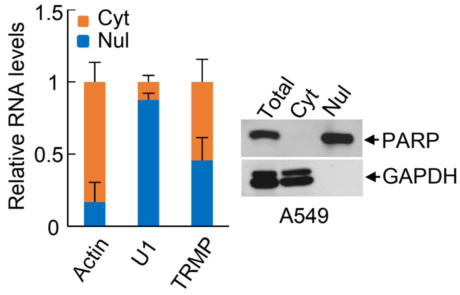

C

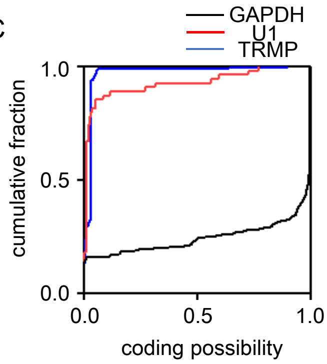

D

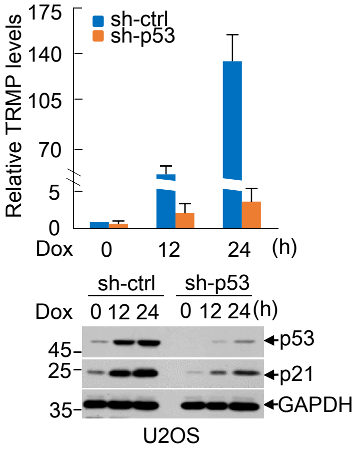

B

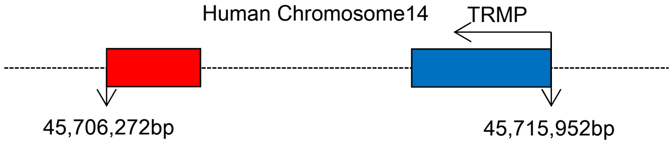

5’TTAGAAGCAGGGAGCTCTCTTCTTCTTCCCC TGGATCC TTCCTTTAAAATAGTT
TCTTTTGTCTTAAGTTTTCATTTCTACGTTTGTCCCTTCATTCAGTCTCTTAAATGA
CGGTCTCAAGCAGTAACAGTAGTAATTGCTGTAATGACGGTCTCAAGTAGTAACC
AGGGCACTCTGCCACAAGTGGCACCTGAACAGGGACTAACAGGG ACTATCAAG
AACAAACGGAGACCTGAAGAGACCTGAAGGGAACTGAAGAGGTCTGCAGGGAC
AAATAGAGATAAGTAGGGATAAATAGAGATAAATAGAGACAAATAGAGATAGGTA
AGGAATGACAGGGACTTG CAGGAAGTAACAGGGACCATAGGGACAGATAGGGA
TAGATAAAGACTAGCAAAGACTAGCGGAGATTAGCAAAGACCAGCAAGGACTAG
CAGAGACTAGCAAAGACTAGCAGAAACTTGCAGGGACAGGTAGAGACAGATAGG
GACAGATAGGGTCCTACAGGGACTTGAACGAGGAAGATCTGCTGGAAAAGAAAA
GAACTACAACCAACCAGACGAACAAGAAACCCTGTTACAAGTCTGC TGGCAGCA
ACATAAGTGACACAAAGTGAACCGGGTTCTATAAATTACCATGGAAAGTGGTGAG
CCAACGATTTGGCAGA ACAAAACCCTGAGGATATGCAAACAAAAGGGACTATGG
AATTTGCAATTGCAGATTAAAATTGAAGAAGAAAACATTTGAGTTGCAAATACCGT
ATTAAAAGTGGACACTGCTTATGACTGCTG TATGCAACAGTTTTTC AGGCCCGA
GGTGAT AAATAAAAAAATTG3’

E

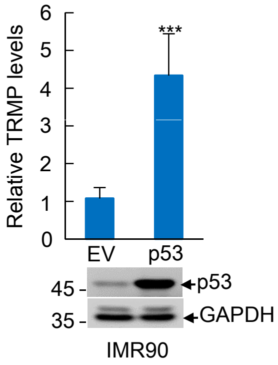

Figure S2

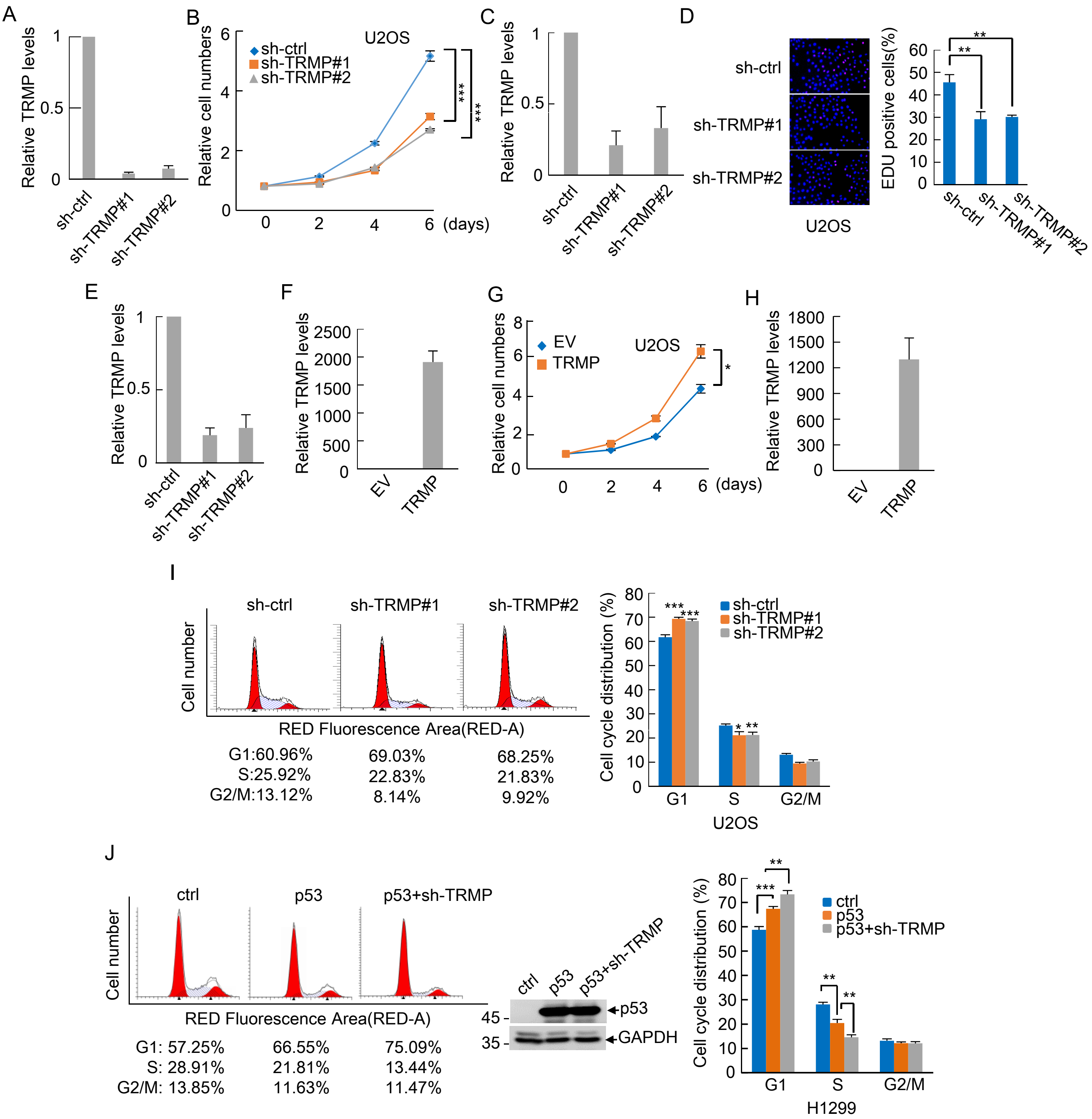
A
Relative TRMP levels
1
0.5
0
sh-ctrl
sh-TRMP#1
sh-TRMP#2
B
Relative cell numbers
U2OS
sh-ctrl
sh-TRMP#1
sh-TRMP#2
***
***
0
2
4
6
(days)
C
Relative TRMP levels
D
sh-ctrl
sh-TRMP#1
sh-TRMP#2
U2OS
EDU positive cells(%)
**
**
E
Relative TRMP levels
F
Relative TRMP levels
2500
2000
1500
1000
500
EV
TRMP
G
Relative cell numbers
EV
TRMP
U2OS
*
(days)
H
Relative TRMP levels
1800
1500
1200
900
600
300
I
sh-ctrl
sh-TRMP#1
sh-TRMP#2
Cell number
RED Fluorescence Area(RED-A)
G1:60.96%
S:25.92%
G2/M:13.12%
69.03%
22.83%
8.14%
68.25%
21.83%
9.92%
Cell cycle distribution (%)
G1
S
G2/M
U2OS
J
ctrl
p53
p53+sh-TRMP
Cell number
RED Fluorescence Area(RED-A)
G1: 57.25%
S: 28.91%
G2/M: 13.85%
66.55%
21.81%
11.63%
75.09%
13.44%
11.47%
45
35
p53
GAPDH
Cell cycle distribution (%)
G1
S
G2/M
H1299

Figure S3

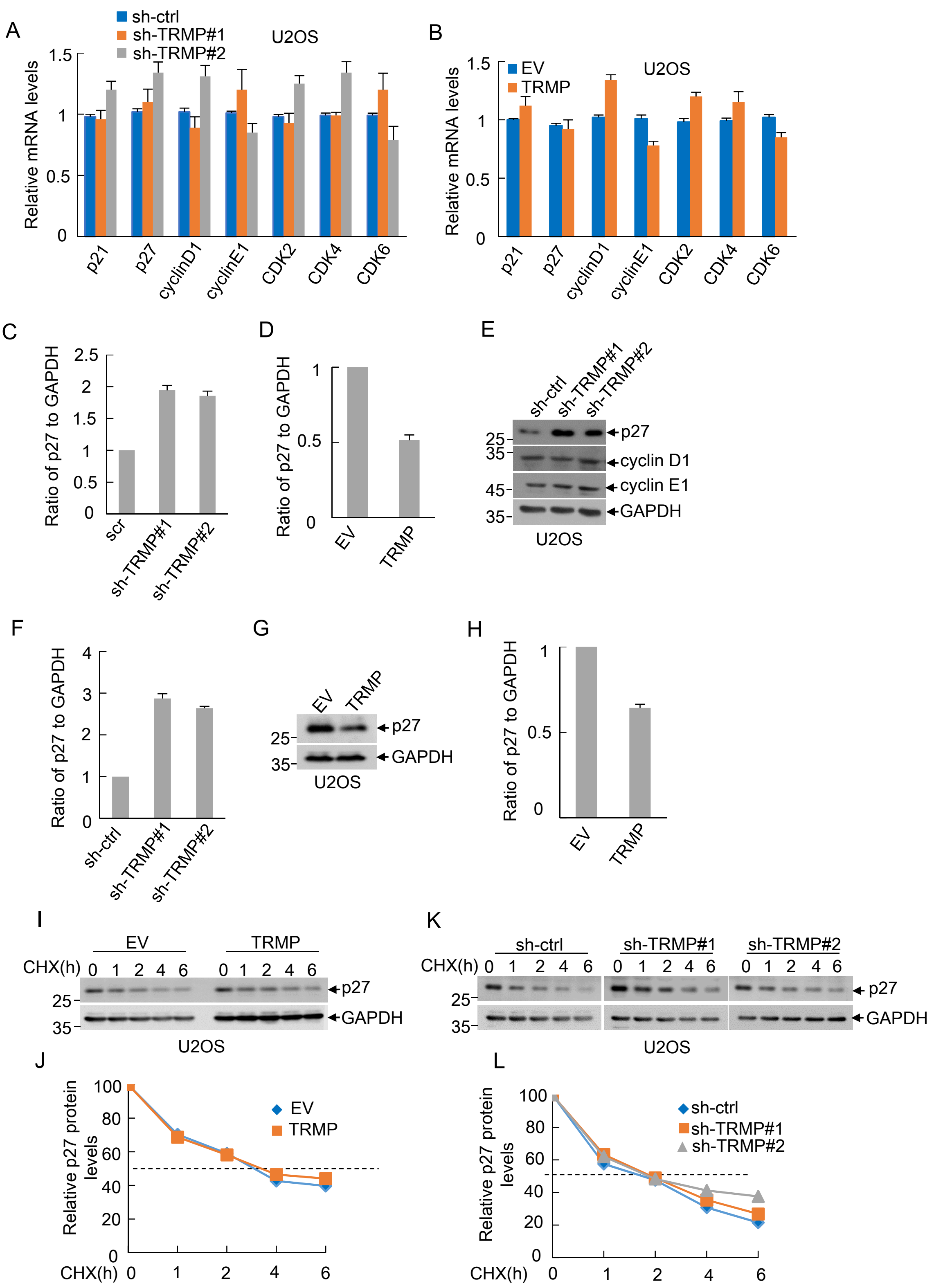

A
sh-ctrl
sh-TRMP#1
sh-TRMP#2
U2OS
Relative mRNA levels
p21
p27
cyclinD1
cyclinE1
CDK2
CDK4
CDK6
B
EV
TRMP
U2OS
Relative mRNA levels
C
Ratio of p27 to GAPDH
scr
sh-TRMP#1
sh-TRMP#2
D
Ratio of p27 to GAPDH
EV
TRMP
E
sh-ctrl
sh-TRMP#1
sh-TRMP#2
p27
cyclin D1
cyclin E1
GAPDH
U2OS
F
Ratio of p27 to GAPDH
sh-ctrl
sh-TRMP#1
sh-TRMP#2
G
EV
TRMP
p27
GAPDH
U2OS
H
Ratio of p27 to GAPDH
EV
TRMP
I
EV
TRMP
CHX(h)
p27
GAPDH
U2OS
K
sh-ctrl
sh-TRMP#1
sh-TRMP#2
CHX(h)
p27
GAPDH
U2OS
J
Relative p27 protein levels
EV
TRMP
CHX(h)
L
Relative p27 protein levels
sh-ctrl
sh-TRMP#1
sh-TRMP#2
CHX(h)

A

The PTBP1 peptide sequences identified by mass spectrometry :

MALIQMGSVEEAVQALIDLHNHDLGENHHLR
AQAALQAVNSVQSGNLALAASAAAVDAGMAMAGQSPVLR
ENALVQMADGNQAQLAMSHLNGHK
NQAFIEMNTEEAANTMVNYYTSVTPVLR
KENALVQMADGNQAQLAMSHLNGHK
IAIPGLAGAGNSVLLVSNLNPER
RGSDELFSTCVTNGPFIMSSNSASAANGNDSK
NNQFQALLQYADPVSAQHAK
GSDELFSTCVTNGPFIMSSNSASAANGNDSKK
NFQNIFPPSATLHLSNIPPSVSEEDLK
DYTRPDLPSGDSQPSLDQTMAAAFGLSVPNVHGALAPLAIPSAAAAAAAAGR
GKNQAFIEMNTEEAANTMVNYYTSVTPVLR
LPIDVTEGEVISLGLPFGK
IIVENLFYPVTLDVLHQIFSK
KLPIDVTEGEVISLGLPFGK
VTPQSLFILFGVYGDVQR
GQPIYIQFSNHKELK
LSLDGQNIYNACCTLR
EGGQEDQGLTKDYGNSPLHR
VLFSSNGGVVK
TDSSPNQAR
DYGNSPLHR
HQNVQLPR
VTNLLMLK
LHGKPIR
SAGVPSR
LTSLNVK

B

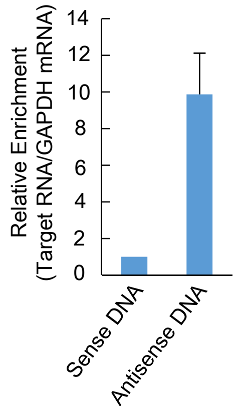

C

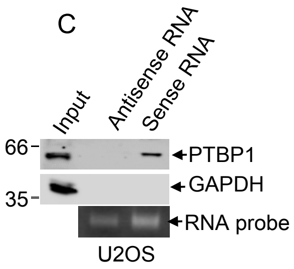

D

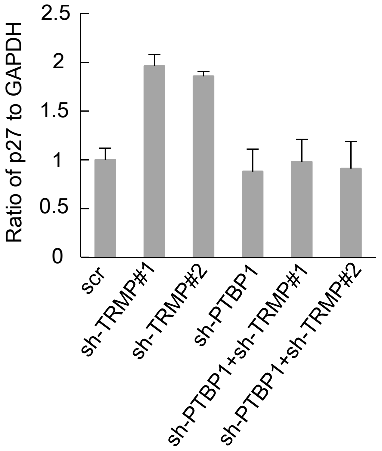

E

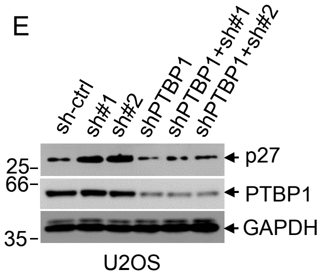

F

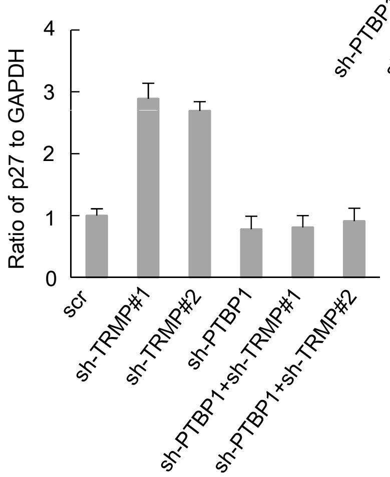

Figure S5

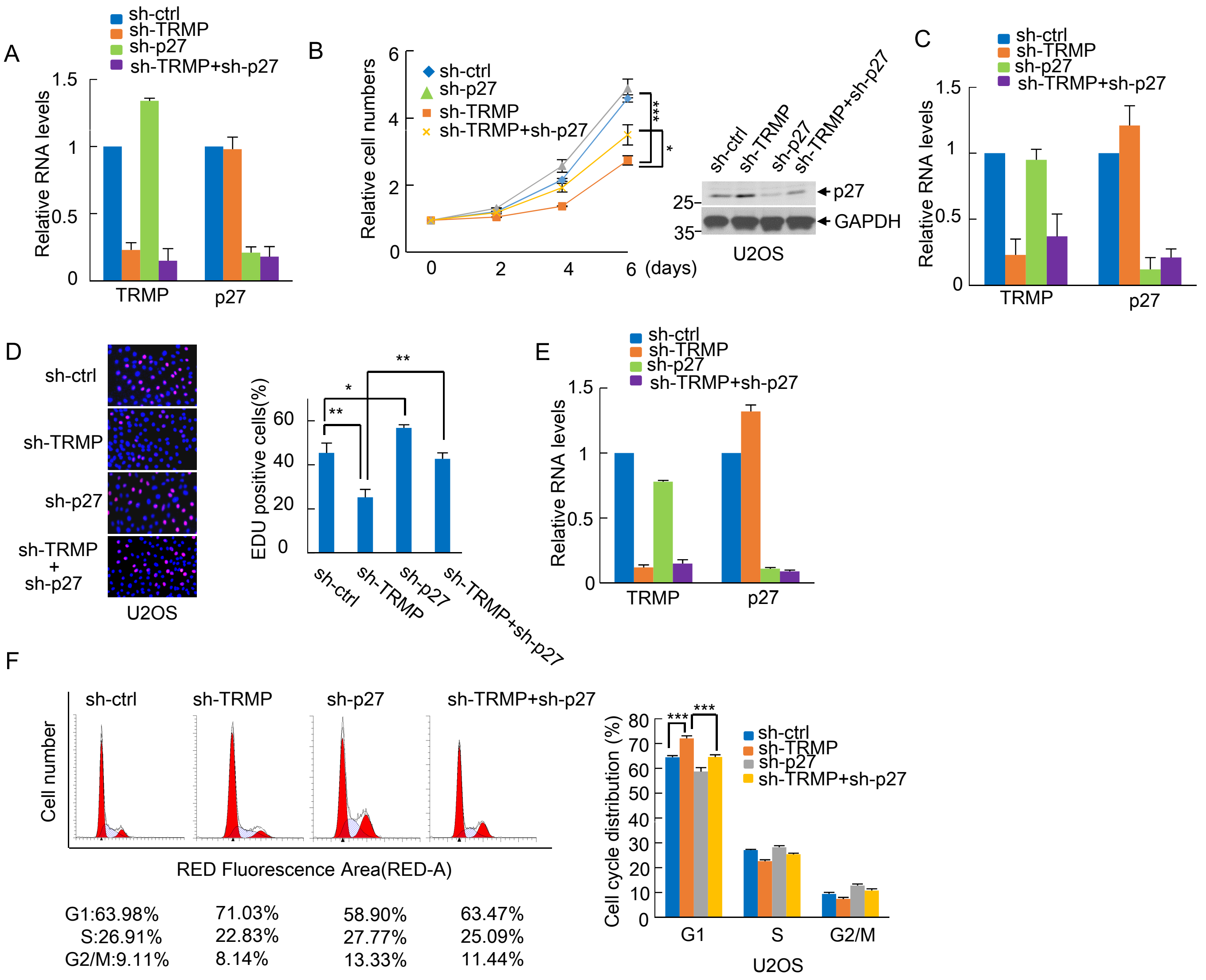
A
sh-ctrl
sh-TRMP
sh-p27
sh-TRMP+sh-p27
Relative RNA levels
TRMP
p27
B
Relative cell numbers
(days)
sh-ctrl
sh-TRMP
sh-p27
sh-TRMP+sh-p27
p27
GAPDH
U2OS
C
Relative RNA levels
TRMP
p27
D
sh-ctrl
sh-TRMP
sh-p27
sh-TRMP + sh-p27
U2OS
EDU positive cells(%)
E
Relative RNA levels
F
Cell number
RED Fluorescence Area(RED-A)
G1:63.98%
S:26.91%
G2/M:9.11%
71.03%
22.83%
8.14%
58.90%
27.77%
13.33%
63.47%
25.09%
11.44%
Cell cycle distribution (%)
G1
S
G2/M
U2OS

Supplementary Table S1

lncRNA	P-value	Absolute Fold change(D+ vs D-)	Regulation(D+ vs D-)	Chromosome	Sequence Name	Gene Symbol	Type
lncRNA-1	1.16423E-09	117.6630069	up	14	ENST00000554810	RP11-369C8.1	noncoding
lncRNA-2	6.82288E-10	109.9187099	up	19	NR_027257	FLJ26850	noncoding
lncRNA-3	3.22E-06	29.34767	up	6	ENST00000437861	RP11-235G24.1	noncoding
lncRNA-4	0.01412	17.07074	up	9	TCONS_00016514	XLOC_007896	noncoding
lncRNA-5	7.24E-06	15.77211	up	X	uc011mhl.1	AK097803	noncoding
lncRNA-6	1.33E-06	14.78519	up	17	NR_026951	LINC00324	noncoding
lncRNA-7	1.86E-06	13.99768	up	9	TCONS_00016338	XLOC_007718	noncoding
lncRNA-8	0.000221	12.69146	up	16	TCONS_00024657	XLOC_011944	noncoding
lncRNA-9	2.22E-08	12.44	up	2	TCONS_00004057	XLOC_001903	noncoding
lncRNA-10	0.002111	10.92624	up	14	ENST00000553613	RP11-7F17.7	noncoding

Supplementary Table S2

Primers used in qRT-PCR assays	
β-Actin	Fw: 5'GACCTGACTGACTACCTCATGAAGAT3'
	Rev: 5'GTCACACTTCATGATGGAGTTGAAGG3'
GAPDH	Fw: 5'CCATGGGGAAGGTGAAGGTC3'
	Rev: 5'GAAGGGGTCATTGATGGCAAC3'
TRMP(lncRNA-1)	Fw: 5' AGTGGTGAGCCAACGATTT3'
	Rev: 5'AGCAGTCATAAGCAGTGTCC3'
lncRNA-2	Fw: 5' AGCTGCGTCTATTTCCTGTG3'
	Rev: 5'TAGACAGTTGCCATGGAGA3'
lncRNA-3	Fw: 5'CCTTCACCTTCCACCATGATT3'
	Rev: 5'TTTGAGAGCAACCGGTTCAG3'
lncRNA-4	Fw: 5' CAAGTATAACTCTTGATTGCCCCGA3'
	Rev: 5'CATGAAAAAGACCAGGATGAAGAC3'
lncRNA-5	Fw: 5'GTCAAGGTAACGTCTGGAGA3'
	Rev: 5'CAGGAGGCTGATGTAGGAAAC3'
lncRNA-6	Fw: 5'ATCCAGGAAAACAGCGATCGA3'
	Rev: 5'CGTCTGTAGACTGGGCATAAC3'
lncRNA-7	Fw: 5'CGGCAATGGATTAATAACAAG3'
	Rev: 5'TAACAGTTGACTTGCCTGA3'
lncRNA-8	Fw: 5'CTTACTGGGGATAGGCCAA3'
	Rev: 5'TGCGAACTCTTTCCATAGAGT3'
lncRNA-9	Fw: 5'GGAAATGCATGATGCTTCCTAAG3'
	Rev: 5'ACCGTGAGTCTTGCTGTAAAT3'
lncRNA-10	Fw: 5' CCACAGAGAGGCACAGCTAAG3'
	Rev: 5' TCGCTTGGCCTGGAAAATCT3'
p21	Fw: 5'TGTCACTGTCTTGTACCCTTG 3'
	Rev: 5'GGCGTTTGGAGTGGTAGAA3'
Cyclin D1	Fw: 5'GTTCGTGGCCTCTAAGATGAA3'
	Rev: 5'AGGTTCCACTTGAGCTTGTT3'
Cyclin E1	Fw: 5'CGGTATATGGCGACACAAGAA3'
	Rev: 5'TGGTGCAACTTTGGAGGATAG3'
p27	Fw: 5'GTCAAACGTGCGAGTGTCTA3'
	Rev: 5'CATGTCTCTGCAGTGCTTCT3'
CDK2	Fw: 5'GTGGCCAGGAGTTACTTCTATG3'
	Rev: 5'AACAAGCTCCGTCCATCTTC3'
CDK4	Fw: 5'ATGTGGAGTGTTGGCTGTATC3'
	Rev: 5'CAGCCCAATCAGGTCAAAGA3'
CDK6	Fw: 5'TGGAGTGTTGGCTGCATATT3'
	Rev: 5'CAACATCTCTAGGCCAGTCTTC3'
**Primers used for in vitro transcription**	
The T7 RNA polymerase sequence(T7) was 5' TAATACGACTCACTATAGGG 3'	
TRMP(sense)	5' (T7）TTAGAAGCAGGG 3'

	5' CAATTTTTATTTATCACCTCGGGC 3'
TRMP(antisense)	5'TTAGAAGCAGGGAGCTCTCTTC3'
	5' (T7）ATCACCTCGGGCC3'
p27-5’UTR	5' (T7)GCCATATTGG3'
	5' CGGCGGCCGCACTCGCACGTTTGAC3'
**Primers used For antisense oligomer affinity pull-down assays**	
TRMP sense oligo DNA	5' (biotin-)TGACACAAAGTGAACCGGGTTCTATAA3'
TRMP antisense oligo DNA	5' (biotin-)TTATAGAACCCGGTTCACTTTGTGTCA3'
**Primers used in ChIP assays**	
P1	Fw: 5' ACATCTCTGGCCTCTATCCA3'
	Rev: 5' TCCCAAAGTGCTGGGATTAC3'
P2	Fw: 5' CTGCTGACCAAATGTGCTTTAC3'
	Rev: 5' GCAGCTATGGAGCAGAAACT3'
GAPDH promoter	Fw: 5' TACTAGCGGTTTTACGGGCG3'
	Rev: 5' TCGAACAGGAGGAGCAGAGAGCGA3'
